# Physician-Reported Practices and Innovation Adoption in Acute Kidney Injury Care in Saudi Arabia: A Nationwide Cross-Sectional Survey: Short title: Acute Kidney Injury Care and Innovation Adoption in Saudi Arabia

**DOI:** 10.31661/gmj.v15i.4260

**Published:** 2026-07-20

**Authors:** Abdulaziz Backer Albacker

**Affiliations:** ^1^ Department of Internal Medicine, College of Medicine, Majmaah University, Al-Majmaah, 11952, Saudi Arabia

**Keywords:** Acute Kidney Injury, Saudi Arabia, KDIGO Guidelines, Biomarkers, Renal Replacement Therapy

## Abstract

**Background:**

Acute kidney injury (AKI) is a major public health concern in Saudi Arabia, where diabetes mellitus, hypertension, sepsis, and critical illness contribute substantially to kidney-related morbidity and mortality. Although international standards for AKI diagnosis and treatment are widely available, national data on physician-reported practice patterns and adoption of emerging diagnostic and therapeutic innovations remain limited.

**Materials and Methods:**

This nationwide cross-sectional survey was conducted from January to June 2025 among physicians involved in AKI care across public, private, and military healthcare facilities in all 13 administrative regions of Saudi Arabia. Participants were recruited through purposive-stratified sampling. The survey assessed physician characteristics, AKI definition and screening practices, current management strategies, biomarker use, artificial intelligence (AI)-enabled tools, telemedicine, emerging therapies, and perceived barriers and facilitators.

**Results:**

A total of 850 of 1,250 invited physicians responded, yielding a response rate of 68%. Most respondents reported using Kidney Disease: Improving Global Outcomes (KDIGO) criteria for AKI definition (82%), fluid optimization protocols (91%), and nephrotoxin-avoidance checklists (88%). Early nephrology consultation within 24 hours was reported by 64% of hospitals and was more frequent in tertiary than secondary centers (78% vs. 45%; P<0.001). Any biomarker use was reported by 37% of physicians, with higher adoption in tertiary centers than secondary centers (52% vs. 18%; P<0.001). Awareness of selected emerging therapies was moderate, but clinical implementation remained below 12%. The main reported barriers were cost (68%), insufficient training (55%), and lack of local clinical guidelines (47%).

**Conclusion:**

Physician-reported AKI management in Saudi Arabia shows substantial alignment with international standards, particularly in preventive and supportive care. However, gaps remain in early nephrology involvement, biomarker implementation, AI-enabled prediction, and equitable access to innovation. National AKI guidelines, targeted training, and structured funding mechanisms may improve early detection, standardize practice, and reduce AKI-related complications.

## Introduction

Acute kidney injury (AKI) is a common and clinically important syndrome associated with increased mortality, prolonged hospitalization, higher healthcare costs, and increased risk of chronic kidney disease (CKD) progression [1, 2]. In Saudi Arabia, the burden of AKI is influenced by the high prevalence of diabetes mellitus, hypertension, sepsis, cardiovascular disease, and critical illness, all of which increase susceptibility to kidney injury [3, 4, 5].

Saudi studies have reported variable AKI frequencies across clinical settings. Hospital-acquired AKI has been described in general and critical-care populations, and AKI prevalence is particularly high among patients requiring mechanical ventilation, patients with community-acquired pneumonia, and critically ill children [3, 4, 6]. Elderly patients are also vulnerable because of multimorbidity, exposure to nephrotoxic medications, and infection-related complications [5].

The Kidney Disease: Improving Global Outcomes (KDIGO) criteria remain the main international standard for AKI definition, staging, and clinical management [1]. Current AKI management is largely supportive and includes optimization of hemodynamics and fluid balance, avoidance or dose adjustment of nephrotoxic medications, monitoring of serum creatinine and urine output, and timely renal replacement therapy (RRT) when clinically indicated [1, 7]. However, delays in AKI recognition and nephrology consultation may contribute to preventable progression and poor outcomes.

Emerging diagnostic and care-delivery technologies may improve early AKI detection and risk stratification. Novel biomarkers, including neutrophil gelatinase-associated lipocalin (NGAL), cystatin C, tissue inhibitor of metalloproteinase-2 (TIMP-2), insulin-like growth factor-binding protein-7 (IGFBP7), and kidney injury molecule-1 (KIM-1), can identify kidney stress or injury earlier than serum creatinine in selected populations [8, 9, 10]. In addition, AI-enabled risk prediction, telemedicine follow-up, and digital health integration may support early intervention and continuity of care, particularly within the context of Saudi Arabia Vision 2030 healthcare transformation [11].

Despite these advances, there is limited nationwide evidence describing physician-reported AKI management practices and innovation adoption across Saudi healthcare facilities. This study aimed to assess current AKI care practices, adherence to evidence-based recommendations, regional and institutional differences in innovation adoption, and perceived barriers and facilitators to implementation among physicians in Saudi Arabia.

## Materials and Methods

### Study Design and Setting

This nationwide cross-sectional survey was conducted from January to June 2025. The target population included physicians who cared for patients with AKI in public, private, and military healthcare facilities across all 13 administrative regions of Saudi Arabia. The study was designed to describe physician-reported AKI practices and innovation adoption rather than to measure patient-level clinical outcomes.

### Participants and Sampling

Physicians were eligible if they had more than two years of clinical experience in AKI management. Physicians with less than two years of relevant experience were excluded. A purposive-stratified sampling approach was used to improve representation across specialties, geographic regions, and hospital levels. The target specialty distribution included nephrologists, critical care physicians, and internal medicine physicians or hospitalists, reflecting the main groups involved in AKI care. The sampling frame included 1,250 eligible physicians identified through professional and institutional directories, including the Saudi Society of Nephrology, Saudi Critical Care Society, and Ministry of Health sources. Hospitals were stratified by geographic region and institutional level to reduce selection bias and improve diversity of practice settings.

### Survey Instrument

The survey instrument included 42 items and was pilot-tested among 50 physicians. Internal consistency was acceptable (Cronbach alpha=0.87). The questionnaire assessed demographic characteristics and practice setting, AKI definition and screening, diagnostic methods, adherence to KDIGO criteria, current management strategies, fluid and nephrotoxin stewardship, RRT timing, biomarker use, AI tools, telemedicine, novel devices or therapies, and perceived barriers and facilitators. Responses were collected using Likert-scale, multiple-choice, and open-ended formats.

### Ethical Approval

Ethical approval was obtained from the King Saud University Institutional Review Board (E-25-4567), with additional local approvals where required. Participation was voluntary and anonymous, and no identifiable patient-level data were collected.

### Statistical Analysis

Categorical variables were summarized as frequencies and percentages. Continuous variables were summarized as mean and standard deviation or median and interquartile range, as appropriate. Between-group comparisons were performed using the chi-square test or Fisher exact test. Multivariable logistic regression was used to identify factors associated with higher innovation adoption according to region, specialty, hospital level, and years of experience. Missing data were handled by listwise deletion when missingness was less than 5%. Statistical significance was defined as P<0.05. The software used for the analyses is IBM Statistical Package for the Social Sciences (SPSS) Statistics version 27.0 (IBM Corp., Armonk, NY, USA).

## Results

**Table:** Table1. Adoption of Innovations by Hospital Level.

Innovation	Tertiary (n=493)	Secondary (n=357)	P-value
Any biomarker	52%	18%	P<0.001*
TIMP-2/IGFBP7	28%	7%	P<0.001*
AI prediction tools	22%	4%	P<0.001*
Telemedicine follow-up	31%	10%	P<0.001*

AI: artificial intelligence; IGFBP7: insulin-like growth factor-binding protein-7; TIMP-2: tissue inhibitor of metalloproteinase-2. ^*^P-value <0.05 is significance.

### Respondent Characteristics

Of 1,250 invited physicians, 850 completed the survey, yielding a response rate of 68%. Most respondents were male (62%). The mean age was 42 years, and the median duration of clinical experience was 12 years (interquartile range, 7-18 years). Respondents were distributed across the Central (32%), Western (25%), Eastern (18%), Southern (12%), and Northern (13%) regions. Tertiary-care centers represented 58% of practice sites, and secondary-care centers represented 42%. Specialties included nephrology (41%), critical care (34%), and internal medicine or hospital medicine (25%).

### AKI Definition, Screening, and Monitoring

Most respondents (82%) reported using KDIGO criteria for AKI definition, whereas 12% used Acute Kidney Injury Network criteria and 6% used Risk, Injury, Failure, Loss, and End-stage kidney disease criteria. Among high-risk patients, including those with sepsis, postoperative status, or contrast exposure, daily serum creatinine monitoring was reported by 71% of respondents, and routine urine output monitoring was reported by 65%.

### Current Management Approaches

Preventive and supportive AKI management was widely reported. Fluid optimization protocols were used by 91% of respondents, nephrotoxic medication avoidance or checklist systems by 88%, and hemodynamic monitoring by 79%. Early nephrology consultation within 24 hours of AKI recognition was reported by 64% of hospitals and was more common in tertiary centers than secondary centers (78% vs. 45%; P<0.001). Supportive care for established AKI included balanced crystalloid fluids in 85% of reported practice settings and vasopressor optimization in 72%. Diuretics were primarily used for volume overload. RRT for stage 3 AKI was initiated by 28% of physicians according to reported KDIGO-compatible indications. Continuous veno-venous hemodiafiltration (CVVHDF) was preferred by 62% of critical care physicians, whereas intermittent hemodialysis was reported by 38%. The most common RRT indications were refractory hyperkalemia (81%), fluid overload (76%), and uremia (69%). The Eastern region reported higher nephrotoxin-stewardship compliance than the national average (94% vs. 88%; P=0.02).

### Outcomes and Challenges

Self-reported in-hospital mortality among patients with stage 3 AKI ranged from 45% to 55%, broadly consistent with local intensive-care unit data reporting mortality ranges of 38% to 51% in advanced AKI settings [3]. Complete kidney recovery before discharge was reported in approximately 42% of survivors. Key challenges included delayed staging, limited access to advanced diagnostic tools in lower-resource settings, and inadequate post-discharge monitoring pathways.

### Emerging Innovations

Biomarker use varied across healthcare settings. Any AKI biomarker use was reported by 37% of physicians. NGAL was the most frequently reported biomarker, used in urine or serum testing by 37% of respondents, followed by cystatin C (24%) and the TIMP-2/IGFBP7 combination test (19%). Biomarker uptake was higher in tertiary centers than secondary centers 52% vs. 18% and odds ratio (OR)=4.2; 95% with 95% confidence interval (CI) is 3.1-5.7) and P<0.001 (Table1). AI-based risk prediction tools were reported by 14% of physicians overall and were more frequently used in academic or tertiary settings. Telemedicine-enabled AKI follow-up was reported by 22% of respondents and was viewed as a potential strategy for improving continuity of care in geographically remote areas. Awareness of emerging therapies, including selective cytopheretic devices, ilofotase alfa or alkaline phosphatase, and RBT-1, was moderate; however, actual clinical use remained below 12%.

### Barriers and Facilitators

The most frequently reported barriers to biomarker and device adoption were high cost (68%), insufficient training (55%), and absence of local clinical guidelines (47%). The most commonly reported facilitators were national protocol development (82%), Ministry of Health funding support (71%), and electronic health record integration (65%). Multivariable logistic regression suggested that nephrology specialty (OR=2.8; 95% CI is 1.2 - 3.4 and P-value is 0.02) also tertiary-care setting (OR=3.9; 95% CI is 1.5 - 3.6 and P-value is 0.03) were associated with higher innovation adoption.

## Discussion

This nationwide cross-sectional survey provides a broad physician-reported overview of AKI management and innovation adoption in Saudi Arabia. The findings suggest that core AKI practices, particularly KDIGO-based definition, fluid optimization, and nephrotoxin stewardship, are widely implemented. However, important gaps persist in early nephrology consultation, biomarker adoption, AI-enabled prediction, telemedicine follow-up, and equitable access to emerging technologies. The reported KDIGO adherence rate of 82% indicates substantial alignment with international standards [1]. Preventive measures such as nephrotoxin avoidance and fluid stewardship are consistent with recommended AKI care bundles and are particularly relevant in Saudi Arabia, where diabetes, hypertension, infection, and nephrotoxic medications contribute to AKI risk [5, 7, 13, 14]. Nevertheless, early nephrology consultation was reported by only 64% of hospitals, with a substantial difference between tertiary and secondary centers. This finding may help explain delays in escalation of care and persistent mortality in advanced AKI. The reported mortality range for stage 3 AKI emphasizes the severe prognosis of advanced kidney injury in hospitalized and critically ill populations. Similar mortality patterns have been reported in local studies of patients with severe infection, intensive-care admissions, and mechanically ventilated community-acquired pneumonia [3, 4, 14]. These findings support the need for earlier identification, structured AKI pathways, and improved transition from inpatient management to post-discharge kidney follow-up. Biomarker adoption was promising but uneven. NGAL, cystatin C, and TIMP-2/IGFBP7 were reported more frequently in tertiary centers, reflecting the influence of laboratory capacity, institutional resources, and subspecialty expertise [8, 9, 10]. The low use of biomarkers in secondary centers suggests a need for cost-effective implementation strategies, including point-of-care testing, standardized pathways for high-risk patients, and targeted funding. The significant difference in innovation adoption by hospital level (Table1) highlights an equity gap that may affect early diagnosis and outcomes. AI-enabled prediction tools and telemedicine follow-up remain early in their implementation. Their adoption is consistent with the broader digital transformation objectives of Saudi healthcare, but uptake remains concentrated in better-resourced centers [11]. Wider implementation will require electronic health record integration, validated local algorithms, clinician training, and governance frameworks addressing safety, accountability, and data quality. The moderate awareness but low clinical use of emerging therapies and devices indicates that innovation adoption is constrained not only by evidence availability but also by cost, procurement, local approval pathways, and clinical training. Reported support for national protocols, Ministry of Health funding, and digital integration indicates that physicians recognize the need for system-level solutions rather than isolated institutional initiatives. Implications for Practice and Policy The findings support development of Saudi national AKI guidelines that adapt KDIGO recommendations to local epidemiology, healthcare resources, and regional disparities. High-risk care bundles should include standardized screening, nephrotoxin review, early nephrology consultation triggers, biomarker pathways where available, and post-discharge follow-up. Training programs through national professional bodies may help improve AKI recognition, RRT decision-making, biomarker interpretation, and AI literacy.

### Strengths and Limitations

The main strengths of this study include nationwide coverage, representation of key specialties involved in AKI care, inclusion of different hospital levels, and a high response rate. The stratified sampling strategy improved representation across geographic and institutional settings and provides useful national-level insight into physician-reported AKI practice. This study also has limitations. First, self-reported data are subject to recall and social desirability bias. Second, mortality and recovery estimates were reported by physicians and were not verified against patient-level clinical databases. Third, the cross-sectional design prevents causal inference. Fourth, some adjusted regression estimates were reported without full confidence intervals and P-values. Finally, the survey assessed reported practice rather than direct adherence measured from clinical records.

## Conclusion

Saudi Arabia shows substantial physician-reported adherence to evidence-based AKI management, particularly in preventive and supportive care. However, gaps remain in early nephrology consultation, biomarker implementation, AI-enabled prediction, telemedicine follow-up, and equitable access to innovation.

National AKI guidelines, structured training programs, funding for diagnostic innovation, and digital health integration may improve AKI detection, standardize management, reduce complications, and support long-term kidney health.

## Conflict of Interest

The author declares no conflict of interest.

## AI Disclosure Statement

The author declare that no AI-assisted tools or services were used during the preparation of this work.
